# Age-related human small intestine methylation: evidence for stem cell niches

**DOI:** 10.1186/1741-7015-3-10

**Published:** 2005-06-23

**Authors:** Jung Yeon Kim, Kimberly D Siegmund, Simon Tavaré, Darryl Shibata

**Affiliations:** 1 Departments of Pathology, University of Southern California Keck School of Medicine, Los Angeles, CA 90033, USA; 2 Preventive Medicine, University of Southern California Keck School of Medicine, Los Angeles, CA 90033, USA; 3 Department of Biological Sciences, University of Southern California, Los Angeles, CA 90089, USA, and Department of Oncology, University of Cambridge, Cambridge, UK

## Abstract

**Background:**

The small intestine is constructed of many crypts and villi, and mouse studies suggest that each crypt contains multiple stem cells. Very little is known about human small intestines because mouse fate mapping strategies are impractical in humans. However, it is theoretically possible that stem cell histories are inherently written within their genomes. Genomes appear to record histories (as exemplified by use of molecular clocks), and therefore it may be possible to reconstruct somatic cell dynamics from somatic cell errors. Recent human colon studies suggest that random somatic epigenetic errors record stem cell histories (ancestry and total numbers of divisions). Potentially age-related methylation also occurs in human small intestines, which would allow characterization of their stem cells and comparisons with the colon.

**Methods:**

Methylation patterns in individual crypts from 13 small intestines (17 to 78 years old) were measured by bisulfite sequencing. The methylation patterns were analyzed by a quantitative model to distinguish between immortal or niche stem cell lineages.

**Results:**

Age-related methylation was observed in the human small intestines. Crypt methylation patterns were more consistent with stem cell niches than immortal stem cell lineages. Human large and small intestine crypt niches appeared to have similar stem cell dynamics, but relatively less methylation accumulated with age in the small intestines. There were no apparent stem cell differences between the duodenum and ileum, and stem cell survival did not appear to decline with aging.

**Conclusion:**

Crypt niches containing multiple stem cells appear to maintain human small intestines. Crypt niches appear similar in the colon and small intestine, and the small intestinal stem cell mitotic rate is the same as or perhaps slower than that of the colon. Although further studies are needed, age-related methylation appears to record somatic cell histories, and a somatic epigenetic molecular clock strategy may potentially be applied to other human tissues to reconstruct otherwise occult stem cell histories.

## Background

**Figure 1:**
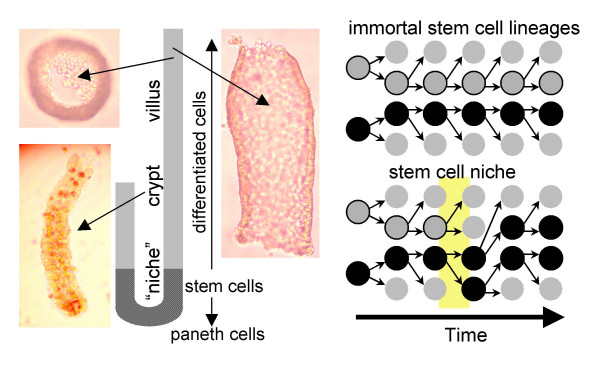
**Schematic of a crypt of Lieberkühn and villus. **Stem cells probably reside within a niche just above the Paneth cells. Differentiated cells migrate out of the niche and up a villus, and die within a week. Illustrated are an isolated crypt and villus fragments. A cross-section illustrates that the villus fragments were essentially hollow tubes of epithelium. The schematic of stem cell lineages illustrates that cell division is always asymmetric (one stem cell and one differentiated daughter) in immortal stem cell lineages, and predominately asymmetric in a stem cell niche. However, niche symmetric divisions occasionally occurs (yellow region) leading to loss of one lineage and the expansion of another, such that total niche stem cell numbers remain unchanged. In this way, niches allow heterogeneous crypts to become homogenous.

The human small intestine is the longest segment of the gastrointestinal tract, approximately five to six meters long in adults. Although it is three to four times longer than the colon, small intestinal tumors are rare, forming about 1% of all gastrointestinal malignancies [1]. Like the colon, the small intestine is composed of multiple crypts (of Lieberkühn), which also contribute cells to finger-like projections called villi (Figure 1). Stem cells appear to reside near the crypt bases, producing numerous differentiated progeny that die within a week as they migrate upwards [2-4]. Stem cells also produce Paneth cells that migrate downwards, and subsequently survive a few weeks.

Although there are likely to be many stem cells per crypt [2-4], intestinal stem cells have not been directly isolated or identified, and their properties and numbers are uncertain. Stem cell lineages may be immortal if at least one daughter retains stem cell properties after each division. However, mouse studies are more consistent with periodic losses of crypt stem cell lineages. The primary observation is that heterogeneous crypts created through mutagenesis or with chimeras eventually become homogenous [5]. If stem cell lineages were immortal, heterogeneous crypts would not become homogeneous.

Stem cell turnover by a niche mechanism [5,6] can explain how heterogeneous crypts become homogeneous (Figure 1). Niche stem cells divide but total niche stem cell numbers remain unchanged as daughter cells constantly leave the niche and differentiate. Usually a stem cell produces one stem cell and one differentiated daughter (~95% of the time in mice [7]), but sometimes a stem cell lineage may become extinct when both daughter cells leave the niche, balanced by another stem cell lineage that expands when both daughters remain within the niche. Such a niche population-type mechanism (random stem cell loss with replacement) eventually results in the loss of all stem cell lineages except one, allowing heterogeneous crypts to become homogeneous (Figure 1).

Human stem cell studies are difficult because the powerful visible fate-marking strategies of animal models are impractical. Most human crypts are visually homogeneous and therefore consistent with either immortal or niche stem cell mechanisms. A study of human colon crypts after therapeutic radiation was consistent with stem cell niches because the frequencies of mutant heterogeneous crypts progressively declined after radiation [8]. Similar studies have not been performed on human small intestines.

One method that can reconstruct the past without direct serial observations infers ancestry from present day sequences. The past is encoded in sequences because errors may accumulate in a clock-like fashion (the molecular clock hypothesis [9]) and record relationships between cells and times since the most recent common ancestor or bottleneck. Just as the age of the human race may be inferred by comparing contemporary genomes, so the ages of crypt stem cell populations may in theory be inferred from their sequences. With immortal stem cell lineages, adult crypts will have greater sequence diversity compared to crypts maintained by stem cell niches that periodically "bottleneck" whenever all but one current stem cell lineage are lost. Therefore, crypt diversity measurements potentially reveal whether stem cell lineages are immortal (last bottleneck around birth) or turn over by a niche mechanism (more recent bottlenecks).

Mutations are rare in normal cells, but recent studies illustrate that stem cell ancestries may be automatically recorded by random somatic epigenetic errors that frequently accumulate with aging [10]. Methylation increases with age at certain CpG-rich regions in the colon [11,12]. This age-related methylation does not resemble a stereotypic developmental process because different cells within the same intestine may have different methylation patterns (5' to 3' order of methylated sites). Such random somatic errors resemble the drift of genomes during species evolution, and by analogy, such drift can be used to reconstruct somatic cell ancestry because methylation exhibits somatic inheritance [13] and all cells are related. Therefore, somatic methylation patterns potentially encode numbers of divisions since birth, and ancestral relationships among cells within a single crypt or villus. Here we infer from methylation patterns that crypt niches containing multiple stem cells maintain the human small intestine.

## Methods

### Specimens

Crypts or villi were isolated from 1–2 cm^2 ^patches of fresh small intestines obtained from 13 patients undergoing surgery for non-small intestinal diseases (Table 1), using an EDTA-containing solution as previously performed for colon crypts [10]. The research was approved by our Institutional Review Board and is in compliance with the Helsinki Declaration. Individual crypts or villus fragments were identified under a dissecting microscope (Figure 1) and placed in 0.5 ml microfuge tubes. Villus fragments were essentially hollow tubes of epithelium without stroma. Nine crypts were analyzed from 13 intestines for CSX, eight crypts from six intestines for MYOD, and 4–10 villi (average of seven) were analyzed for CSX from six intestines. The DNA was extracted and bisulfite converted as previously described [10]. DNA was also extracted from microdissected paraffin-embedded small intestinal tissues of an eight month old and a four year old. The bisulfite treated DNA from about half a crypt was amplified by PCR at CpG-rich segments of CSX or MYOD [10]. PCR products were cloned into bacteria and eight clones were sequenced from each crypt or villus.

**Table 1:** Specimens

Patient	Age/Sex	Site	Surgery	Specimen	Tags
A	17/F	Ileum	Constipation	Crypt	CSX, MYOD
B	27/F	Ileum	Inflammatory Bowel	Crypt, Villi	CSX
C	46/F	Ileum	Ovarian Cancer	Crypt, Villi	CSX, MYOD
D	51/M	Duodenum	Whipple	Crypt	CSX
E	52/M	Ileum	Colorectal Cancer	Crypt, Villi	CSX
F	58/F	Duodenum	Whipple	Crypt	CSX, MYOD
G	58/F	Ileum	Colorectal Cancer	Crypt,Villi	CSX, MYOD
H	63/F	Duodenum	Whipple	Crypt	CSX
I	67/F	Duodenum	Whipple	Crypt	CSX, MYOD
J	72/M	Ileum	Colorectal Cancer	Crypt	CSX
K	75/F	Ileum	Colorectal Cancer	Crypt,Villi	CSX
L	76/M	Ileum	Colorectal Cancer	Crypt	CSX, MYOD
M	78/M	Duodenum	Whipple	Crypt,Villi	CSX
N	0.07/F	Ileum	Obstruction	Paraffin	CSX, MYOD
O	4/M	Ileum	Trauma	Paraffin	CSX, MYOD

Ileum specimens were proximal portions of total colectomies.

### Stem cell dynamics from methylation patterns

Small intestinal methylation patterns were analyzed by the same quantitative approach we applied to colon crypt methylation patterns ([10], and its Supplementary Materials). Human crypts of Lieberkühn containing a total of ~500 cells [14] were simulated on a computer, modeling the processes of stem cell division (symmetrical or asymmetrical), differentiation, death, and random methylation errors. The parameters of the model (Table 2) are numbers of stem cells per crypt, probabilities of symmetric or asymmetric stem cell division, a methylation error rate, and numbers of divisions since birth. These parameters are uncertain for the human small intestines, and the values are just guesses chosen to be consistent with the biology of the intestines, and are similar to the values we estimated for the colon [10]. We emphasize that they are just estimates, and further experiments are needed to define these values better. The model starts with unmethylated sites in the CpG-rich regions of CSX or MYOD, and errors are assumed to accumulate independently between CpG sites with the same rates as simulated in the colon [10]. The output is the methylation patterns of these regions after a given number of divisions. A total of 1000 simulations were performed for each crypt scenario.

**Table 2:** Model Parameters

Parameter	Stem Cell Niche	Immortal Lineages
Stem Cells per Crypt	4–256	2
Probability of Asymmetric Stem Cell Division (P1)*	0.98 to 0.89	1.0
Probability of Symmetric Stem Cell Division: zero (P0) or two (P2) stem cell offspring*	0.02 to 0.11	0
Methylation Error Rate	2 × 10^-5 ^per CpG site per division**	2 × 10^-5 ^per CpG site per division**
Stem Cell Division Rate	0.75 per day	0.75 per day

*P0+P1+P2 = 1, with P0 = P2

** Equal probabilities for methylation and demethylation errors

A stem cell hierarchy is simulated with small numbers of stem cells that produce differentiated mitotic cells, which subsequently become differentiated non-mitotic cells. For example, if there are two stem cells per crypt, differentiated mitotic cells divide six more times before becoming non-mitotic. Differentiated non-mitotic cells remain through two more divisions with the oldest cells dying after each division cycle to maintain a constant number of crypt cells. In this scenario, there are 512 cells per crypt – two stem cells, 126 differentiated mitotic cells, and 384 differentiated non-mitotic cells. With greater numbers of stem cells, numbers of differentiated mitotic cells and their divisions are correspondingly reduced to maintain a constant size of the mitotic compartment and 512 cells per crypt.

Immortal stem cell lineages were simulated with strictly asymmetric division producing one stem cell and one differentiated daughter (P1 = 1.0). After a given number of stem cell divisions, eight alleles are randomly sampled from each simulated crypt, as in the experimental approach. Niche and immortal scenarios were identical except niche stem cells also exhibit symmetric divisions (P1 < 1.0). Total niche stem cell numbers remain constant because divisions that produce two differentiated daughters are balanced by divisions that produce two stem cell daughters (P0 = P2). Symmetric divisions tend to reduce crypt diversity because methylation patterns can be lost through lineage extinction.

The Paneth cell compartment was not specifically modeled because its dynamics are uncertain and these cells would be rarely sampled because their relative numbers are small (~2–4 Paneth cells per crypt section [15]). Paneth cell methylation patterns should be similar to those of other differentiated cells because they survive only a few weeks in mice [16], although human Paneth cells may potentially have different lifetimes.

Methylation of CSX or MYOD "tags" can be converted into a 5' to 3' binary code, with "0" representing an unmethylated site and "1" representing a methylated site. For example, an unmethylated CSX tag (eight CpG sites or 256 different possible tags) is "00000000" and a fully methylated MYOD tag (5 CpG sites or 64 different possible tags) is "11111". Tags can be summarized with three statistics that reflect numbers and types of stem cell divisions:

1) Percent Methylation. Numbers of methylated tag sites can be summarized by percent methylation. For example the CSX tag "01010101" is 50% methylated. In general, percent methylation reflects numbers of divisions since birth.

2) Unique Tags per Crypt. Diversity, or numbers of unique tags among the eight tags sampled from a crypt, reflects numbers of stem cells and stem cell lineage survival. Greater numbers of stem cells or longer-lived stem cell lineages would lead to greater crypt tag diversity.

3) Intracrypt Distance. This is the average number of site differences between crypt tags (Hamming distance). For example, the distance between CSX tags 00000011 and 11000000 is four. Intracrypt distance is another measure of crypt diversity.

## Results

**Figure 2:**
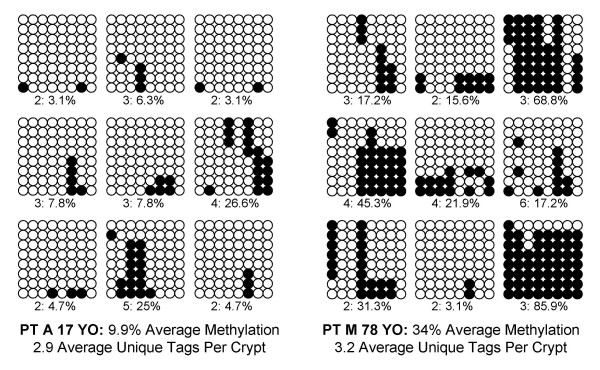
**Examples of CSX crypt methylation patterns. **Each group represents eight CSX tags (5' to 3' horizontal orientation, each of the eight CpG sites is represented by a circle) sampled from a single crypt, with nine crypts per patient. Open circles are unmethylated sites and filled circles are methylated sites. Numbers of unique tags per crypt and crypt percent methylation are summarized below each crypt. The seemingly random methylation patterns collectively encode both numbers of divisions since birth and ancestry.

**Figure 3:**
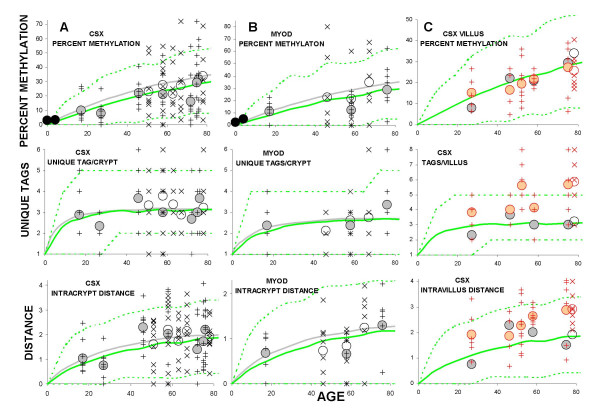
**Methylation tag analysis**. **A: **Summary of CSX methylation tags sampled from crypts of Lieberkühn. Individual crypt values ("X" for duodenal crypts and "+" for ileal crypts) and average values (open circles for the duodenum and filled circles for the ileum) were plotted for 12 small intestines. Black filled circles represent the methylation measured in fixed small intestine from children. Solid green lines represent average values for a stem cell niche simulation best matching the experimental data. Consistent with the scatter of experimental crypt values, niche simulations also produced a variety of different outcomes summarized by dotted green lines representing 95% of simulated outcomes. Gray solid lines represent average simulated values consistent with the colon [10]. Small intestine percent methylation appeared to increase more slowly than the colon with age, consistent with fewer stem cell divisions relative to the colon. **B: **Summary of MYOD crypt of Lieberkühn methylation tags. **C: **Summary of CSX villus methylation tags. Average crypt values (black circles) for the same intestine are also plotted for comparison with average villus values (red circles). Unique tags and distances were significantly greater in villi compared to crypts whereas percent methylation was not significantly different.

Crypts of Lieberkühn and villus fragments were isolated from fresh human small intestine (Figure 1) and individual allele methylation patterns were analyzed by bisulfite sequencing. Examples of crypt CSX methylation patterns (5' to 3' order of methylated sites) or "tags" are illustrated in Figure 2. Methylation represents somatic errors because CSX tags, like most CpG-rich sites [13], are initially unmethylated at birth. Although the tag patterns were complex, with differences within crypts and between crypts in the same intestine, the average percent methylation generally increased with age (Figure 3A). Crypt diversity (unique tags per crypt and intracrypt tag distances) also exhibited age-related changes (Figure 3A). Duodenum and ileum tag patterns appeared similar.

Different tags within the same intestine are consistent with stochastic errors that accumulate independently in different cells. To extract ancestral information encoded by seemingly random tags within a single intestine, crypts were modeled assuming stochastic methylation errors and either immortal or niche stem cells (see Methods). The primary difference between the models is that the probability of symmetric division (yielding two stem cell (P2) or two differentiated daughters (P0)) is zero with immortal stem cells, whereas the probability of symmetric division is greater than zero with niche stem cells (Table 2). Greater inter-crypt variability is expected with stem cell niches because both methylation errors and stem cell survival are stochastic.

**Figure 4:**
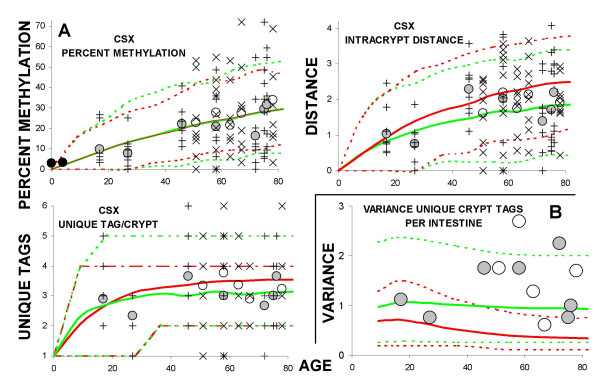
**Niche versus Immortal Stem Cells**. **A: **Comparisons between niche (green) and immortal (red) stem cell simulations with CSX tags. Niche simulations assume a constant number of stem cell (from 4 to 256 stem cells per niche) and probabilities of asymmetric division (P1) from 0.98 to 0.89 (see Table 3). Outcomes of all niche simulations were similar and therefore one cannot be certain of the exact stem cell niche size. Immortal lineage simulations assume two stem cells per niche. Parameters (see Table 2) were otherwise identical between the simulations. Outcomes were more variable with niche simulations with wider 95% simulation intervals (dotted lines). The differences are most obvious with unique tags per crypt. **B: **Variances of unique CSX crypt tags per intestine (for example from Figure 2, Patient A had 2, 2, 2, 2, 3, 3, 3, 4, and 5 unique tags per crypt for a variance of 1.1, and Patient M had 2, 2, 2, 3, 3, 3, 4, 4, and 6 unique tags per crypt for a variance of 1.7). Simulations with the immortal lineage scenario (average is a solid red line, 95% intervals are dotted lines) were less consistent with the experimental data (circles) than the niche simulations (green lines).

As in the experimental data, the simulated crypts contained tags with different patterns, summarized by averages and intervals including 95% of simulated crypt outcomes (Figure 4A). Average simulated crypt values were consistent with average experimental values for either two immortal stem cells per crypt, or 4 to 256 niche stem cells. However, niche stem cells produced more inter-crypt tag variation, which better matched the variability observed in the small intestines. Inconsistent with immortal stem cell lineages were the observations that unique crypt tag variances were greater than simulated average values for all 12 intestines, and only 4 of the 12 intestines were within 95% simulation intervals for immortal stem cells (Figure 4B). In contrast, experimental variances for 10 of 12 intestines fell within 95% simulation intervals for the niche scenarios. In addition, more than four unique sequences per crypt were often observed, which should occur only rarely with two immortal crypt stem cells (four possible alleles), but may occur more frequently with 4 to 256 niche stem cells (Figure 4A).

**Figure 5:**
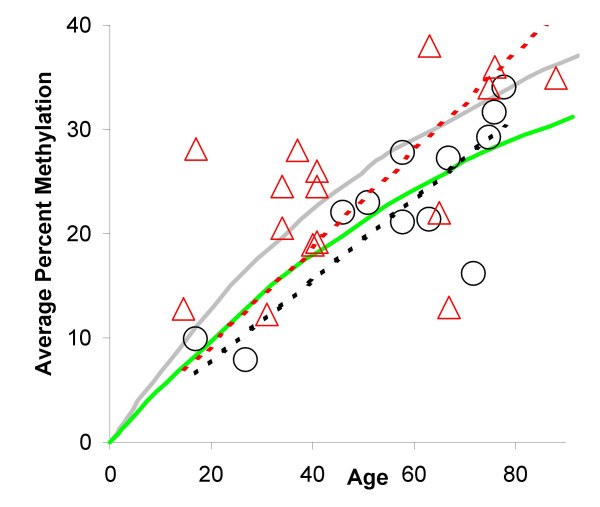
**Comparisons of large and small intestine age-related CSX methylation rates**. Average percent methylation increased more slowly with age in the small intestine (black circles) relative to previously published [10, 17] colon measurements (red triangles). However this trend (black and red dotted lines) was not significant. A lower stem cell division rate was more consistent with the small intestinal data (green line, 0.75 divisions per day), relative to a model [10] postulating one division per day for the colon (grey line).

Tag data from human large [10] and small intestinal crypts were similar, except the percent methylation appeared to increase more slowly with age in the small intestine (Figure 3A). Small intestine percent methylation was generally lower than published [10,17] measurements of human colon crypts, with a trend towards a slower increase with age (Figure 5), but this difference was not significant (p = 0.24, F-test). Potentially methylation error rates are lower in the small intestine, but assuming equivalent error rates (a molecular clock hypothesis [9]), stem cell division rates are either equivalent or lower in the small intestine compared to the colon. For example, simulations with a mitotic rate 75% of the colon (0.75 divisions per day) and a methylation error rate identical to the colon (2 × 10^-5 ^per site per division) better fit the small intestine data (Figure 3A and Figure 5).

Crypt methylation patterns encode niche dynamics, but exact niche stem cell numbers and probabilities of symmetric divisions remain uncertain because different combinations yield similar outcomes (Table 3). These niche combinations (4–256 stem cells and P1 from 0.98 to 0.89) are the same in large [10] and small intestines. For example, assuming that 95% of stem cell divisions yield one stem cell daughter, the crypt of Lieberkühn niche size is 64 stem cells. As in the colon [10], loss of all niche stem cell lineages except one or a "bottleneck" would recur on average every 3,000 divisions (with a 95% interquantile range of 1,000–7,000 divisions). Small intestinal crypt diversity (unique tags per crypt) did not change with age (Figure 3A), suggesting that niche dynamics or numbers of niche stem cells do not change with aging.

MYOD tags sampled from six small intestines were also consistent with the same niche parameters inferred with the CSX tags (Figure 3B). For a further test of our model, CSX tags were sampled from villi of six intestines. Villi should exhibit more tag diversity than crypts because they receive contributions from four to ten surrounding crypts [18]. Furthermore, if methylation reflects the lifetime accumulation of stem cell mitotic errors, percent methylation should be similar between villi and crypts because crypt cells are only a few divisions older than villus cells. Consistent with these expectations (Figure 3C), there were significantly more unique tags per villus than per crypt (average 4.8 versus 3.0, p < 0.001, two-tail t-test) whereas methylation differences were not significant (average 21.8% versus 22.9%, p = 0.76). Distances between tags within a villus were also significantly greater than tag intracrypt distances (2.5 versus 1.7, p <0.001). Villus and crypt comparisons illustrate the ability of methylation tags to encode simultaneously both mitotic age (numbers of divisions since birth) and distinctly different ancestries.

**Table 3:** Stem Cell Niche Parameters

Niche Size	P asymmetric (P1)*	P symmetric (P0+P2)
4	0.98	0.02
16	0.97	0.03
64	0.95	0.05
256	0.89	0.11

*P0+P1+P2 = 1, with P0 = P2

## Discussion

**Figure 6:**
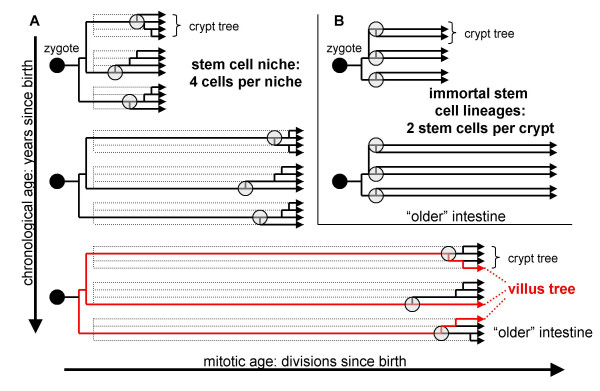
**Somatic cell trees**. **A: **A niche somatic cell tree. Starting from the zygote, millions of related cells create an intestine. Within each crypt are multiple stem cells (a niche with four stem cells is illustrated). With age, branches become longer reflecting more divisions since birth. Most niche stem cell lineages are lost (dotted lines) from random stem cell loss with replacement, which continuously creates newer more recent common crypt ancestors (circles). Such common crypt ancestors are stem cells because all current crypt cells are progeny of such cells. Any one of the four current niche stem cells may become a common ancestor, but only one will. In this way, crypts can appear to have both a single stem cell (most recent common crypt ancestor) and multiple potential stem cells. A villus tree (red lines in bottom tree) has essentially the same age as the crypt tree (same number of divisions since birth) but villi should exhibit more diversity than crypts because villus cells have older most recent common ancestors. **B: **Somatic cell tree with immortal stem cell lineages. Stem cells always divide asymmetrically and their lineages never become extinct. Unlike a niche somatic cell tree, branches are never lost or "pruned".

The adult small intestine is physically composed of millions of crypts, and cells that constantly divide and die. Underlying the homeostatic visual appearance of a normal small intestine is an ancestral somatic cell tree that grows with age (Figure 6). Starting with the zygote, millions of related cells create an intestine. Past and present cells are all connected through time by ancestral lineages or branches, which ultimately root in the zygote or first common ancestor. Although stem cell definitions may differ [2-4], a crypt common ancestor is equivalent to a stem cell because all cells in a clonal crypt unit are progeny of this single common stem cell ancestor.

Both immortal and niche stem cell mechanisms can theoretically produce appropriate numbers of differentiated cells consistent with the morphology of normal small intestine. However, the shape of an intestinal somatic cell tree differs whether stem cell lineages are immortal or maintained by niches (Figure 6). Immortal stem cell lineages result in multiple long crypt branches, with the most recent common crypt ancestor present around birth. In contrast, a niche mechanism constantly creates newer more recent common crypt ancestors as most niche stem cell lineages become extinct.

Methylation tags can distinguish between immortal or niche stem cell lineages because random somatic epigenetic errors will accumulate differently. Specifically, a niche mechanism produces more variability between crypts (wider variation in unique tags per crypt within a single intestine) and less intracrypt diversity (smaller average intracrypt distances) than immortal stem cells (Figure 4). Small intestine and colon crypt methylation patterns [10] were similar and more consistent with stem cell niches. The same stem cell dynamics (Table 3) fit both colon and small intestinal niches. There were no apparent differences between ileal and duodenal crypt niches, and niche stem cell numbers (like the colon [10]) did not appear to change or decline with age.

Niche stem cells defined by ancestry are physically intangible because common ancestors are defined by past events and no longer exist. Stem cells with immortal lineages are more readily identified because their pasts and futures are predictable. However, niche stem cell fates are unpredictable because all may potentially become common ancestors but only one will. The inability to predict niche survival may help explain why some adult stem cells have been so difficult to isolate or characterize. A niche somatic cell tree has few branches because random stem cell turnover eventually "prunes" all niche lineages except one (Figure 6). Such bottlenecks are predicted by our analysis to recur on average after 3,000 divisions.

Other mechanisms may also be consistent with crypt methylation patterns and the assumptions of our model remain unverified [19]. For example, the observed variability in methylation patterns could also be generated if stem cell numbers were different between crypts within an intestine [19]. Although our bisulfite sequencing appears technically adequate (no evidence of nonconversion of C to T and only rare mutations (<1 per 1,000 bases) at non-CpG sites), it is difficult to check the accuracy and reproducibility of the data. Repeat sampling of a crypt typically yields similar but not identical methylation patterns (data not shown), which may reflect crypt heterogeneity or experimental artifact.

One strategy to test empirically a stochastic model that inherently yields scattered results is to examine similar but biologically distinct entities. Both small intestine and colon crypts are thought to be maintained by stem cell niches [5,6] and the successful application of our model to the small and large intestines with methylation patterns from CpG-rich sequences on different chromosomes (CSX and MYOD) is empirical support for its ability to infer stem cell dynamics. In addition, small intestinal villi are physically different from crypts and represent mixtures from multiple adjacent crypts. Villi should be more diverse than crypts, yet villi and crypts should have similar numbers of divisions since birth. As one would expect if methylation records somatic cell histories, villus and crypt tag percent methylation were not significantly different, and villi contained significantly more tag diversity than crypts. Therefore, methylation patterns and our model are consistent with colon and small intestine crypt niches, and small intestine villi. Genetic alterations also appear to modulate stem cell dynamics because certain germline APC mutations are associated with significantly more diverse tags in normal-appearing familial adenomatous polyposis colon crypts, consistent with increased niche stem cell survival [17].

Mouse studies suggest that stem cell division rates are higher in the small intestines than the colon [2,3,20]. However, it is difficult with most assays to distinguish between stem and non-stem cell proliferation, and there are few human small intestine stem cell studies. Of interest, neoplasia, as with Min mice [21], is more frequently observed in the murine small intestines relative to the colon, whereas human small intestinal tumors are a small fraction of colon tumors [1]. In contrast to murine studies, human small intestinal stem cell division rates appeared to be slower or similar to those in the colon because age-related methylation appeared to increase more slowly in the small intestine (Figure 5), although the difference was not significant. Assuming a molecular clock hypothesis [9] or equivalent CSX methylation error rates throughout the lower gastrointestinal tract, the data are more consistent with equivalent or slightly lower small intestinal stem cell division rates relative to the colon.

**Figure 7:**
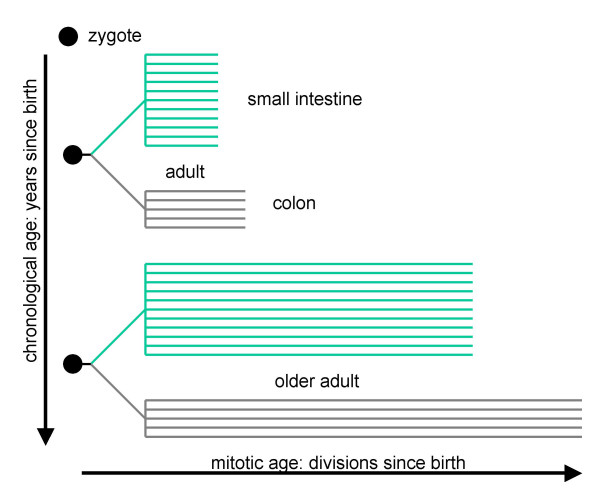
**An intestinal tree based on mitotic ages or numbers of divisions since birth**. Each horizontal branch reflects a single crypt. Although all tissues have the same chronological ages, differences in stem cell division rates allow tissues within the same individual to have different mitotic ages. A small intestine tree has a more crypt branches (greater numbers of crypts) than a colon, but its branches may be shorter, which can possibly help explain why despite the greater physical size of the organ, small intestinal tumors are infrequent relative to colonic ones.

There is no definitive evidence supporting a somatic cell molecular clock hypothesis, but a priori a slower small intestinal stem cell division rate is more consistent with human intestinal cancer epidemiology [1], because fewer small intestinal errors (genetic or epigenetic) would be expected to accumulate during aging. A slower small intestinal stem cell division rate could also help explain why crypt purification is faster in the murine colon relative to the small intestine [5]. Moreover, an ability to count relative divisions since birth in different human tissues allows distinctions (Figure 7) between mitotic tissue ages (divisions since birth) and chronological ages (years since birth). The colon and small intestines within an individual have equivalent chronological ages but may have different mitotic ages.

## Conclusion

Methylation patterns suggest that niches containing multiple stem cells maintain crypts throughout the lower gastrointestinal tract. Small intestine stem cells appear to divide at equivalent or slower rates relative to the colon, and niche dynamics remain stable during aging. Although methylation tags only indirectly track stem cell dynamics and their exact interpretations are uncertain, they potentially allow for the systematic investigation of any intestine without prior experimental intervention. A somatic cell tree must underlie all human tissues and studies based on the hypothesis that certain somatic methylation errors record somatic cell histories may better define how cells divide and die during normal and abnormal aging.

## Competing interests

The author(s) declare that they have no competing interests.

## Authors' contributions

JYK performed most of the experiments and helped write the manuscript. KDS and ST helped with the statistical and quantitative analysis. DS analyzed the data and wrote the manuscript.

## Pre-publication history

The pre-publication history for this paper can be accessed here:


